# Patients’ coping strategies in the first 2 years of rheumatoid arthritis: a qualitative study

**DOI:** 10.1016/j.ero.2025.11.008

**Published:** 2025-12-05

**Authors:** Stina Larsson, Ingrid Larsson, Elisabeth Mogard

**Affiliations:** 1 School of Health and Welfare, Halmstad University, Halmstad, Sweden; 2 The Swedish National Road and Transport Research Institute (VTI) Linköping, Sweden; 3 Department of Behavioural Sciences and Learning, Linköping University, Linköping, Sweden; 4 Spenshult Research and Development Centre, Halmstad, Sweden; 5 Department of Clinical Sciences Lund, Rheumatology, Lund University, Lund, Sweden; 6 Department of Rheumatology, Skåne University Hospital, Lund, Sweden

## Abstract

**Objectives:**

Rheumatoid arthritis (RA) is a chronic disease that requires lifelong medication and self-management. Few studies have explored the coping strategies patients use to make life manageable after an RA diagnosis, and how these strategies function in the adaptive process of living with RA. The aim was to describe coping strategies patients with RA use to manage everyday life during the first 2 years after diagnosis.

**Methods:**

A purposeful sample of 22 patients, each with a disease duration of 12 to 20 months, participated in interviews conducted either in focus groups or individually. The study used an abductive qualitative content analysis combining inductive identification of coping strategies with deductive categorisation.

**Results:**

The analysis revealed various coping strategies and their functions, which were categorised into 10 subcategories and 3 categories. They represented the adaptive processes used by patients to coordinate actions and contingencies within their environment, rely on available social resources, and align preferences with available options.

**Conclusions:**

During the early years with RA and modern medical care, patients expressed the need to use various coping strategies to manage everyday life. The strategies varied depending on the patient’s life situation and context and ranged from making a mental effort, to accepting and adjusting their mindset, to planning and finding solutions.

**WHAT IS ALREADY KNOWN ON THIS TOPIC**

• Coping strategies are essential for patients with chronic diseases, such as rheumatoid arthritis (RA), to manage their daily lives.
• Previous studies mainly focus on established RA, showing that maladaptive coping is linked to poorer mental health.

**WHAT THIS STUDY ADDS**

• Identifies diverse coping strategies used by patients within the first two years after RA diagnosis.
• Categorizes coping strategies into three adaptive processes: mangaging and adapting actions, relying on social resources, and aligning preferences with options.
• Highlights both problem-focused (planning, problem-solving) and emotion-focused (acceptance, avoidance) strategies.

**HOW THIS STUDY MIGHT AFFECT RESEARCH, PRACTICE OR POLICY**

• Increases awareness among clinicians of coping strategies in early RA, including maladaptive forms.
• Supports person-centred care to strengthen adaptive coping and prevent long-term psychosocial difficulties.
• Provides a foundation for developing multidisciplinary interventions that enhance coping in early RA.

Alt-text: Unlabelled box

## INTRODUCTION

Rheumatoid arthritis (RA) is a chronic inflammatory systemic disease affecting approximately 0.5% to 1% of the population worldwide [1]. It is more prevalent in women than in men, regardless of age [2]. As a chronic disease, RA can significantly impact various aspects of life, including daily routines, professional commitments, interpersonal relationships, and emotional well-being [3]. RA may also have socioeconomic consequences, particularly if patients no longer can work full-time [1,4]. Moreover, pain, fatigue, physical disabilities, and the risk of developing deformities can impair patients’ quality of life [1,5]. Approximately 40% to 60% of patients with RA experience severe fatigue, which negatively affects not only quality of life but also level of activity, and social relationships [[6], [7], [8], [9]]. In addition, anxiety and depression can be consequences of the disease, as well as triggers for worsening conditions [10]. A systematic review found that the prevalence of depression among patients with RA ranges from 15% to 48%, depending on the screening tool used [11].

Early diagnosis and prompt treatment with conventional and biological disease-modifying antirheumatic drugs, along with nonpharmacological treatment options, are essential for patients with RA. The treat-to-target approach is a recommended strategy for monitoring pharmacological treatment outcomes, with the goal of achieving remission [12]. Despite the medical advances made, there remain several unmet needs [1], which are diverse and evolve over time as the disease progresses [13]. Person-centred care is essential, because various treatment outcomes, potential side effects, and patient preferences need to be considered [3,14]. Patients with recently diagnosed RA have expressed a need for contact with a multidisciplinary team, which is considered an important component in person-centred care [15]. Involving patients in shared decision making about their treatment and care is also crucial for boosting motivation and fostering adherence and may ultimately improve overall health [3,5]. Patients’ expertise regarding their situation extends beyond clinical tests, and their perspective should therefore complement clinical research [14,16,17]. In addition, person-centred care with a holistic perspective [3] has been shown to improve the detection of mental health problems, comorbidities, and disease outcomes [18]. Due to the fact that RA significantly affects all aspects of a patient’s life, it is important to incorporate their occupation and living conditions into the care planning process. A person-centred approach ensures that patients are equipped with the necessary tools and support to manage their lives while coping with RA [3].

Patients with RA experience various symptoms and often require lifelong treatment; therefore, it is crucial to explore the coping mechanisms they use when dealing with the stress associated with the challenges and internal, emotional responses linked to their condition [19]. Coping encompasses the diverse ways patients manage life’s stressors and obstacles, including the emotions these challenges evoke [19]. Coping strategies include behaviours, perceptions, and cognitive processes that extend beyond disease-specific situations [20]. Their effects may be observed in both the short and long term, contributing to improved mental and physical well-being [21]. Research has shown that coping strategies form an integral part of addressing the disease’s consequences, such as pain and mental health issues, which significantly impact a patient’s overall quality of life. Coping strategies are influenced by factors like the disease’s severity and the patient’s age [22]. Furthermore, the time elapsed since diagnosis plays a vital role in shaping a patient’s subjective perception of the disease’s severity, including psychological attributes such as coping strategies [23]. In patients with established RA, a previous meta-analysis found that maladaptive coping (including emotional avoidance and passive coping) in contrast to a more adaptive coping style, with problem-focused strategies, is associated with increased psychological distress [24]. A study in women with established RA found that those who move towards more acceptance regarding their RA-related challenges and limitations increased their problem-solving skills and regained control in life [25]. Men with established RA experience challenges in managing life, but they also face challenges regarding their masculinity, roles, and abilities [26]. In early RA, previous research has indicated that the adoption of negative coping strategies, such as feelings of injustice, bitterness, and isolation, is associated with psychological challenges [27], and it has been observed that insufficient or negative coping strategies, in conjunction with pain-related issues, can contribute to psychosocial challenges that extend beyond the experience of pain [28]. In addition, previous research has emphasised the importance of clinical awareness of various coping strategies and the need to identify and support patients with early RA and maladaptive coping strategies [[22], [23], [24], [25], [26], [27]]. Given the above-mentioned findings and the importance of adaptive coping strategies to manage chronic disease, it is essential to increase healthcare professionals’ knowledge and awareness of the coping strategies used by patients with RA during the early phase of the disease. To the best of our knowledge, there is a gap in qualitative research focusing on coping strategies used by patients during the first years with RA. Therefore, this study aimed to describe the coping strategies used by patients with RA to manage their everyday lives during the first 2 years after diagnosis.

## METHODS

### Design

This study used qualitative content analysis with an abductive approach. Qualitative content analysis focuses on subjects and contexts, aims for variation, and is used to identify patterns and recurrent categories. An abductive approach implies moving between inductive and deductive reasoning. This approach not only enabled closeness to the empirical data but also allowed the findings [29] to be related to Skinner’s model of coping strategies [21]. In addition, it provided a structure for understanding how the respondents used coping strategies to manage everyday life and their impact on the process of adapting. Reporting in this study was carried out in accordance with COREQ-36 guidelines [30].

### Participants

The study used a purposeful sample of 22 patients with RA comprising 15 women and 7 men, who were interviewed within the first 2 years after diagnosis (12-20 months) (Table 1). In this study, sex refers to biological distinctions (female/male); gender was not assessed. Patients were recruited from a regional rheumatology clinic and a university hospital in southern Sweden and invited to participate in the study by their nurse or rheumatologist. The inclusion criteria were an RA diagnosis according to the American College of Rheumatology/European League Against Rheumatism 2010 criteria [31], an age of 18 years or over and the ability to write, speak, and understand Swedish. Thirty-one patients who previously had taken part in individual interviews 3 to 7 months after diagnosis were invited to participate in the study. Of those, 9 declined to participate and interviews were conducted with the remaining 22 approximately 1 year later, as a follow-up. The inclusion criteria were based on time since diagnosis, although it should be noted that many patients experience symptoms prior to formal diagnosis, which means that their lived experience of RA may extend beyond the diagnostic timeframe. Participants’ characteristics are presented in Table 1.

**Table 1:** Participant demographic and self-reported health characteristics

Variables	Participants (N = 22)
Sex (n)	
Female	15
Male	7
Age (y)	57 (42-81)
Civil status (n)	
Cohabiting	19
Living alone	3
Educational level (n)	
Primary	5
Secondary	11
University	6
Employment (n)	
Employed	12
Retired	10
NRS pain (mm)^a^	20 (0-50)
NRS general health (mm)^a^	25 (0-95)
NRS fatigue (mm)^a^	40 (0-100)
Disease duration (mo)	18 (12-21)
DMARD treatment duration (mo)	17 (12-20)
Current RA treatment (n)	
csDMARDs	16
bDMARDs	6
Discontinued treatment	3

bDMARD, biological disease-modifying antirheumatic drug; csDMARD, conventional synthetic disease-modifying antirheumatic drug; DMARD, disease-modifying antirheumatic drug; NRS, numerical rating scale.

Values are median (range) if not otherwise stated.

a During the past week, ranging from 0 (best) to 100 (worst).

### Data collection

Data collection took place in 2018 and involved interviews with 22 patients. The interviews were organised into 7 focus groups, each comprising 2 or 3 patients, while an additional 5 patients were interviewed individually. The focus groups and individual interviews were performed by 2 rheumatology nurses with no previous relationship with the patients. One of the researchers (IL) has extensive experience in qualitative methods and rheumatology, while a nurse in rheumatology with some experience in qualitative methods conducted the interviews under IL’s supervision. The focus groups were held in a private room without interruptions, either at the clinics or at a research and development centre, and the individual interviews were conducted over the phone or face-to-face in a private room according to the patients’ preferences. The initial ambition was to conduct focus group interviews to capture interaction and shared meaning making. As 5 patients could not or did not want to participate in a group setting, individual interviews were also conducted. Using the same interview guide for both formats ensured comparability, while the 2 approaches provided complementary insights. The overall focus of this study was ‘Coping with everyday life in the first 2 years after diagnosis’. An interview guide with open-ended questions was used, including questions about changes in patients’ lives after diagnosis, the impact of RA on their everyday lives, and strategies for coping with RA. Probing questions were used, such as: ‘Please, tell me more about…’, ‘Could you clarify what you mean?’ or ‘What do you have in mind when you say …?’ The focus groups lasted between 54 and 110 minutes, with a median duration of 78 minutes. The individual interviews lasted between 20 and 58 minutes, with a median duration of 35 minutes. All the interviews were digitally recorded and transcribed verbatim.

### Data analysis

The data analysis followed the steps of qualitative content analysis and was conducted in an abductive manner. This means that the researchers moved iteratively between inductive identification of meaning units and codes and deductive interpretation guided by an existing model [29]. The data analysis started with an inductive phase, in which the interviews were read multiple times to ensure familiarity with the content, with a search for meaning units reflecting the patients’ coping strategies. The meaning units were condensed and coded, where the codes represented coping strategies. The analysis continued with a deductive phase in which the codes–in this study, the coping strategies–were sorted into families of coping according to their functions and desired outcomes. The concept of families was used to show that they represent higher-order categories and are multidimensional and multifunctional [20]. The structure used is an adaptation of Lazarus model, in which the organisation of the coping families serves as an attempt to bridge the gap between coping strategies and adaptive processes [21]. In this study, the model by Skinner et al [21,32] was slightly adapted: the categories were referred to as the adaptive processes, while the family functions in the adaptive processes, explained by families of coping, constituted the subcategories (Table 2) [21]. Adaptive processes describe how an individual adapts after a stressful event. In the structure by Skinner et al [21], there are 12 families of coping strategies, of which 10 were detected in our interviews with patients. The 2 missing families not found in the interview data were helplessness and delegation [32].

**Table 2:** The subcategories describe the families of coping and their function in the adaptive process. The categories describe the adaptive processes used by patients with RA, which were adapted to the model by Skinner et al [21].

Adaptive processes (categories)	Coordinate actions and contingencies in the environment	Coordinate reliance and social resources available	Coordinate preferences and available options
Family function within the adaptive process (subcategories) —Family of coping	Adjust actions to be effective through Problem-solving Plan for additional contingencies through Information seeking Escape noncontingent environments through Avoidance	Protect available social resources through Self-reliance Use available social resources through Support seeking Withdrawal from unsupportive context through Isolation	Flexibly adjust preferences to options through Accommodation Find new options through Negotiation Give up preferences through Submission Remove constraints through Opposition

In this study, the concept of coping strategy is used synonymously with family of coping as defined by Skinner et al [21]. The analysis was performed by SL (social worker), while IL (rheumatology nurse, professor in health and lifestyle, and experienced researcher in qualitative methods) and EM (physiotherapist in rheumatology) acted as co-assessors. The analysis was discussed multiple times in the research group until consensus was reached, to ensure that the interpretation was credible and confirmable and accurately represented the data.

### Ethical approval

The study was carried out in accordance with the ethical principles of the Declaration of Helsinki (World Medical Association) and was approved by the Regional Ethical Review Board in Lund, Sweden (nos. 2016/618, 2017/205). All participants received written and oral information about the study, participated voluntarily, and could withdraw at any time without providing a reason and without affecting their health care and treatment in any way. The data were processed according to the General Data Protection Regulation.

## RESULTS

The results showed that, during the first 2 years with RA, patients used various coping strategies to deal with everyday life and illness. The coping strategies and their functions in the adaptive process (subcategories) were sorted within the adaptive process itself; in this study, the categories described the adaptations to life after diagnosis. These adaptative processes were used to coordinate patients’ actions with contingencies in the environment, coordinate patients’ reliance on others with available social resources in the environment, and coordinate patient’s preferences with available options in the environment.

### Coordinate actions and contingencies in the environment

The coordination of actions and contingencies within the environment refers to the ability of patients, during the first 2 years after their RA diagnosis, to manage and adapt their actions and responses based on the circumstances and challenges present within their surroundings. Patients expressed that they used various coping strategies, including problem-solving to adjust actions to be more effective, information seeking to plan for additional contingencies, and avoidance to escape noncontingent environments.

### Adjust actions to be effective

The patients expressed that they adjusted their actions to be effective through problem-solving. They also adjusted their behaviour to address problems and used numerous coping strategies to adapt their actions to living with a disease that can make life unpredictable and unstable. Coping strategies, such as problem-solving, planning, strategising, and carrying out different types of instrumental actions were used to adjust activities in patients’ professional and everyday lives. Some patients realised they needed to plan everyday life and activities differently after being diagnosed with RA. In contrast, others explored different options for managing their symptoms, such as easing pain and fatigue or keeping their energy levels up throughout the day. For some, this could be achieved by taking pauses or naps during the day, while for others, these strategies were not always enough.

‘It has taken over my entire life because you plan according to it [the disease]. If I do this, how much energy do I have left?’ (Focus Group 3)

The patients engaged in physical activities as both short- and long-term coping strategies to feel better and maintain their capacities over time. The results showed an awareness of the importance of movement. The patients used the coping strategy of instrumental action, whereby they prioritised physical activities aimed at improving health, maintaining functional abilities, and preventing decline. Some of the patients used training equipment that they had been prescribed.

‘There are tools that can help you, especially for the hands. I think it’s great because you really want to get back to what you had’. (Individual Interview 2)

Patients took responsibility by making long-term strategic decisions and using various coping strategies to preserve their functional capacities, enhance their abilities, and optimise everyday life through effective action adjustments.

### Plan for additional contingencies

The patients described how they attempted to plan for additional contingencies through information seeking. They actively searched for more information or resources to gain more control, learn more about the disease and the treatment, being prepared for unforeseen situations, and to map possibilities during their initial years with RA. The patients said that they acquired information by reading online, requesting advice from healthcare professionals with experience in rheumatology or talking to other patients with RA.

‘They [healthcare professionals] are really good at what they do. … they give you advice …, it’s really good. I have had a lot of use for them’. (Focus Group 3)

The results also reflected participants’ frustration when healthcare professionals did not provide the information they needed. One way they coped with this was to search for and read information more independently, primarily online, to ensure that all relevant information was obtained. Another coping strategy was to consult with other patients or healthcare professionals. In cases where the need for information was not met, the patients experienced their health care as unpredictable and insufficient.

‘Information, really …. Now, when I got new medicine, it didn’t work, so I got another type, and then I received information from a nurse and we sat down and talked. That’s what I should have got from the beginning’. (Focus Group 3)

### Escape noncontingent environments

The results also include coping strategies of an avoidant nature. Patients described how they sought to avoid situations that did not offer viable solutions. These coping strategies were used when patients strove to avoid a noncontingent environment faced after being diagnosed with RA, leading to mental withdrawal, avoiding thoughts about the disease or disliking situations that reminded them of the disease or their limitations. Patients reported internal strategies, such as cognitive avoidance and denial, as ways to distance themselves from the disease, which made them feel less sick. Some admitted to avoiding thoughts about the disease to create this mental distance. Others expressed that they did not like to be reminded of their condition because they were trying their best to forget about it.

‘As I perceive it, the more you talk about it and the more you think about it, the sicker you get, in some way. Because I’m not ill in my world’. (Individual Interview 3)

Behavioural avoidance was another coping strategy patients used, whereby they would postpone or stop engaging in certain activities due to, for example, increased fatigue.

‘You need to rest more. So, I’ve changed my area of work, and I had to make some changes. Because my body couldn’t bear it’. (Focus Group 5)

These coping strategies are aimed at planning and adjusting everyday life by adapting actions to be effective, improving or maintaining skills to influence both the present situation and the future, searching for information to plan for contingencies, and escaping or avoiding a noncontingent context. Avoidance strategies are often driven by emotions such as fear and the instinct to flee, whereas strategies involving activity and initiative are often motivated by curiosity, interest, and a desire to master new situations.

### Coordinate reliance and social resources available

Patients described a variety of coping strategies to protect and use their available social resources and withdraw from an unsupportive context to coordinate reliance and social resources available.

### Protect available social resources

The patients protected their available social resources through a variety of coping strategies and described that they were self-reliant and regulated their emotions and behaviours. Self-reliance was noticeable when the patients expressed their opinions and feelings about medication and made independent adjustments to their treatment. Some patients even questioned the doctor’s knowledge and authority, confident in their abilities to make changes based on their own knowledge. Other patients preferred cooperation with healthcare professionals in managing and taking responsibility for their treatment. For these patients, it was crucial to trust both the healthcare professional’s knowledge and competence and the treatment plan. By regulating their emotions regarding the disease and the treatment, patients fostered feelings of safety and confidence in their situation, especially if they experienced positive results from the treatment.

Respondent 1: ‘I don’t like cortisone’.

Respondent 2: ‘Haha, I don’t think anyone likes it, but by God, it’s great when you need it’. (Focus Group 5)

Self-reliance could also manifest itself as behavioural regulation, such as striving to live a healthy life. Some patients had changed their diet on their own initiative in an effort to become healthier.

### Use available social resources

Using social resources was another type of coping strategy described by the patients. They used coping strategies such as seeking contact, comfort, and instrumental aid from others, which involved obtaining both active physical and social support. These coping strategies provided feelings of social connection, security, and reduced loneliness.

‘I think it’s important no matter what disease you have. If you feel you have a lot of support and get help, that will make you feel better faster…’ (Individual Interview 4)

The patients stated that the social resources could include family, friends, or healthcare professionals from the multidisciplinary team and that a spectrum of assistance was desired, ranging from moving heavy pots to receiving social support from relatives or aid and support from competent healthcare professionals. When healthcare professionals had enough time and competence, were accessible, and showed involvement and interest, the patients felt reassured.

### Withdrawal from an unsupportive context

When social resources were not considered supportive, patients sometimes used the coping strategy of social withdrawal. Withdrawing and avoiding others led to changed patterns in life.

‘…I am less social because I’m tired. I postpone things’. (Focus Group 7)

These coping strategies were shaped by an environment that was unable to support the patient’s needs.

### Coordinate preferences with available options

Patients described coping strategies aimed at coordinating personal preferences with available options as a way of managing thoughts about the future. In response to the challenges of living with RA, they expressed flexibility by adjusting or giving up their preferences, identifying new possibilities, and removing constraints in light of their changed conditions.

### Flexibly adjust preferences to options

Some of the patients used mental coping strategies that involved adjusting their preferences to the options they considered realistic. This was evident in cognitive restructuring, wherein they realised the need to change their thinking, given that RA is a chronic disease requiring lifelong treatment and self-management. Patients described how this translated into aspiring for greater acceptance, maintaining a positive mindset, and improving their ability to stay present in everyday life.

‘But … I know I will never be as healthy as I was before and that’s sad … you have to accept it. … I should be glad; twenty years ago there was hardly anything that could help. That was harder’. (Individual Interview 2)

Minimisation was also used as a coping strategy. This was observed when patients recounted instances during which they pushed themselves to engage in activities despite feeling tired or experiencing pain. Reasons for this behaviour could include social obligations or a desire not to miss out on social activities. Trying to remain active, socially engaged and continuing to enjoy hobbies were also ways in which they safeguarded their quality of life.

### Find new options

The patients described several coping strategies they used to adjust to their changed life situations. These included negotiation, bargaining and priority setting to adjust to life and find new options going forward. Negotiating strategies focused on problem-solving and exploring new options. For example, difficulties in getting enough sleep due to symptoms like pain and discomfort were addressed by using pillows and adjusting body positioning instead of relying on medicines. Despite showing some resistance, patients also used aids as a compromise between their previous, desired, and current methods of coping with RA.

‘I have a raised seat on my toilet. And I bought one of those knives, but I couldn’t use it. I couldn’t get used to it, I want to cut the usual way’. (Focus Group 6)

### Give up preferences

Rumination and expressions of grief and worry could be directly linked to the coping strategies patients with RA used to deal with their new life situation. Patients expressed that they had felt compelled to give up previous life priorities due to their impaired physical functions and stated that they needed to process these losses and manage the impact of these changes on their current everyday life situations.

‘It’s more like, finally you accept the situation, because I didn’t do that straight away. … you have to get used to it and figure out how to make the most of the situation’. (Focus Group 5)

The patients stated that talking, sharing thoughts, and receiving information were helpful elements in the process of ceasing rumination and trying to feel better. Not only did the participants vocalise the importance of meeting and talking to others, some participants in the individual interviews also expressed that they wanted opportunities to meet other patients with RA.

### Remove constraints

The patients showed varying degrees of resistance towards the limitations caused by RA and pharmacological treatment. Coping strategies aimed at removing constraints by opposition appeared in statements in which they described how they made their own assessments and departed from their treatment plans or changed physicians’ multiple times because of their discontent.

‘It [the pharmacological treatment] could result in problems with the eyes as well, … I did it on my own volition, just stopped taking it, because I find it really hard to get in touch with the rheumatologist here’. (Focus Group 1)

Other coping strategies that emerged can be interpreted as a willingness to compromise and find new options, to regain a sense of control. For example, some patients temporarily abandoned prescribed treatments but sought to negotiate with healthcare professionals regarding alternative approaches. Not all coping strategies stemmed from flexibility; some arose from rigidity, anger, and defiance. This was manifested in questioning the healthcare professionals’ knowledge or by expressing suspicion of treatments.

## DISCUSSION

The findings indicate that during the initial years following a diagnosis of RA, patients used diverse coping strategies to navigate environmental demands and contingencies, while simultaneously negotiating a balance between personal autonomy and reliance on available social resources. They also used different coping strategies to coordinate their preferences with available options in relation to their changed life situation. Patients perceived that the symptoms and consequences of RA, especially fatigue and pain, affected everyday life and that this required the implementation of various strategies such as problem-solving, planning, and strategising. They described how they adapted their actions to everyday life by, for example, planning breaks and naps to be able to stay alert and active throughout the day. In previous research, patients with RA have described fatigue as an overwhelming, unpredictable, and consuming symptom that impacts not only activities and work but also psychosocial and emotional factors and their quality of life [33,34]. The patients in our study described how they used both short-term and long-term coping strategies to manage different symptoms and consequences of the disease. Patients’ experience of fatigue has been shown to worsen even when disease activity and other symptoms decrease, making relief from fatigue increasingly important for patients over time [17]. From the patient’s perspective, it is important that health professionals address fatigue and its impact on patients’ everyday life, activities, and quality of life [6,35] and offer support to facilitate effective coping strategies, when needed. Recently, it has been reported that severe fatigue is related to use of negative cognitive emotion regulation strategies by patients with RA [36]. In our study, more long-term strategic decisions that patients made manifested as trying to preserve their capacities and improve their abilities.

Information seeking was described as an important way to learn more about the disease and plan for additional contingencies. In addition to written information, patients appreciated getting advice and well-timed information from knowledgeable healthcare professionals and other patients. This aligns with research on patients’ perceptions of patient education, indicating that patients want to be engaged and make informed choices about managing their disease and care [37]. However, if patients were dissatisfied with the information or found it insufficient, they sometimes expressed frustration, and a way to cope with this was to search for information by themselves.

Some patients used cognitive avoidance to try to forget their disease, to feel better, and to continue their everyday life as before. Previous research has shown the importance of treating patients with respect and not labelling them as just ‘patients’ in health care interactions [17]. Person-centred care is an approach that addresses this issue by focusing on the person behind the patient and tailoring care based on the patient’s needs, preferences, and resources [38,39]. This approach fosters a more respectful and empathetic health care environment that recognises each person’s unique experiences and contributions [40,41]. In optimal care, patients are active partners who collaborate in co-creating their health care [41,42]. People usually resist being treated as objects or solely patients, which is part of a functional pattern of resisting [43]. As the present results show, resistance can manifest itself as mental and behavioural withdrawal, avoidance of situations in which patients are reminded of their disease, or denial. In previous research, avoidance has also been shown to be a common coping strategy among patients with early RA, manifesting itself as the avoidance of activities, movement, social interactions, and sometimes even medication [44,45]. When studying coping strategies, it is important to consider the time elapsed since diagnosis, to understand if coping strategies are functional and have more long-term effects [21].

The patients in this study emphasised their need for regular contact with health care professionals and to be recognised as experts in their own situations. They also described coping strategies centred on self-reliance. Previous research has shown that it is common for patients to prefer to take control of their disease, treatment, and life [34] as this can enhance empowerment and self-management and thereby strengthening patient’s role within health care [40,41]. Studies have also shown that patients may have ambiguous feelings towards their pharmacological treatment and sometimes take the initiative to adjust doses and frequencies more independently [5]. The findings in our study showed that patients cope by seeking comfort and support from both health care professionals and their friends and relatives. Effective and supportive communication between patients and health care professionals is crucial to achieving optimal care [14,40,41]. Seeking support and comfort from friends and relatives has previously been reported as important in encouraging the use and effectiveness of coping strategies by patients [5,40,41]. Patients with RA expressed that they would appreciate forums where they can discuss the disease’s psychological impact and receive support on how to deal with, for example, fatigue, emotions, work and spare time, relationships, and malaise [14]. To foster the development of effective coping strategies like self-reliance and support seeking, successful rheumatology care should incorporate early interventions, consistent follow-up and personalised holistic approaches [46].

Another coping strategy identified in the results was minimisation, which research indicates serves as a protective factor that significantly impacts daily life, by lessening the impact of a situation, emotion, or behaviour. Minimisation is associated with fewer psychosocial issues, suggesting it can help mitigate some of the disease’s negative effects [27], and may enable patients to align their preferences with changing conditions. Previous research shows that living with RA can lead to a significant shift in identity, particularly for men, since reduced strength and abilities challenge their masculine roles and sense of independence [26]. Loss of power and control over one’s body can result in feelings of helplessness, anger and frustration, and affect various aspects of life. The concept of normality can also shift as the disease progresses, prompting a reassessment of what constitutes a normal life [17]. This adaptive process is reflected in the study’s findings, where patients cope by coordinating preferences with available options through negotiation, aiming for acceptance and prioritising new goals. Patients with early RA have previously been shown to use acceptance strategies to cope with the disease, including accepting the pain, adjusting to a slower work pace and allowing for more time to complete tasks [44,45].

The results identified 10 of the 12 families of coping strategies [32] used by patients during the first 2 years after their RA diagnosis. The 2 missing families were helplessness, which includes strategies like confusion, cognitive interference or cognitive exhaustion, and delegation, which includes strategies like complaining, whining, and self-pity [32]. This absence may be explained by the fact that patients had been diagnosed 12 to 21 months prior to the study, suggesting that they may have developed a more resilient mindset over time [13]. No delegation-related coping strategies were mentioned, such as maladaptive help-seeking, complaining, whining, or self-pity. These strategies have previously been associated with greater disease severity [22], which could partly explain their absence in our study, as the patients received modern rheumatology care and could contact team members in the multiprofessional team when needed.

In qualitative research, trustworthiness is defined by 4 criteria: credibility, dependability, confirmability, and transferability [29,47]. The credibility of this study was strengthened using well-established research methods. All results were included without omission, which enhances credibility [47]. Additionally, using both individual interviews and focus groups demonstrates the use of complementary methods. Focus groups facilitate diverse perspectives and discussions, while individual interviews may be better suited to participants who are uncomfortable sharing their views in a group setting [48]. Recruitment through clinical staff, may have influenced the composition of the sample towards patients with regular contact with health care and higher engagement in their treatment, which should be considered when interpreting the findings. Dependability was increased by maintaining consistency across all interviews by using the same questions [47]. Follow-up questions were used to clarify and avoid misunderstandings, while participants were encouraged to express themselves openly during the interviews. The involvement of a multidisciplinary research team in the analysis process further strengthened the study’s dependability. Confirmability was ensured by carefully reporting the analytical steps and providing detailed descriptions of the participants’ experiences, which are supported by substantiating quotations [29]. The interviews were rich and varied, capturing diverse experiences comprehensively. To strengthen transferability, purposeful sampling [47] included participants from university hospitals and regional settings, ensuring variation in age, sex, and living location. The sample size (n = 22) included 15 women and 7 men, reflecting the higher prevalence of RA among women. As 9 of the invited patients declined participation, some perspectives may not have been captured, which could have limited the variation of coping experiences represented. Overall, the level of participation (22 of 31 invited) ensured that a wide range of experiences was included. Some limitations should be noted. The use of both focus groups and individual interviews may have influenced the type of data obtained, although the same interview guide was used in both formats and the data were considered comparable and complementary. Another limitation is the time gap between data collection and reporting; changes in health care, including those related to the COVID-19 pandemic, may have shaped coping strategies, although the process of coping with a chronic illness such as RA is unlikely to have fundamentally changed. Finally, as symptoms may precede formal diagnosis, coping strategies may reflect a longer lived experience of RA than the diagnostic timeframe indicates. Overall, the experiences described in this study likely reflect those of patients with RA in the first 2 years after diagnosis and may be transferable to similar clinical settings.

### Conclusions

This study describes how patients with RA used a range of coping strategies during the first 2 years after their diagnosis to manage the ongoing challenges faced in everyday life. These coping strategies ranged from mental efforts to accept and adjust their mindset to planning, rearranging daily activities, finding solutions, and maintaining their physical capacities. Patients with RA expressed how they sought information, invited others to help them, and relied on their own judgement. Some strategies included avoidance and ignoring the disease and its consequences. The coping strategies varied depending on each patient’s context and life situation, thus highlighting the need for holistic care that addresses individual needs, context, personality, and lifestyle. Awareness of patients’ coping processes is essential for health care professionals in rheumatology care to provide person-centred care and effective support for patients.

## CRediT authorship contribution statement

**Stina :** Writing – review & editing, Writing – original draft, Methodology, Formal analysis, Conceptualization. **Ingrid Larsson:** Writing – review & editing, Supervision, Project administration, Methodology, Formal analysis, Conceptualization. **Elisabeth Mogard:** Writing – review & editing, Supervision, Methodology, Funding acquisition, Formal analysis, Conceptualization.
